# Supplementary material for: Phylogenetic Characterization of Crimean-Congo Hemorrhagic Fever Virus Detected in African Blue Ticks Feeding on Cattle in a Ugandan Abattoir
Source: Microorganisms. 2021 Feb 20;9(2):438. doi: 10.3390/microorganisms9020438 (PMC7923759; doi:10.3390/microorganisms9020438)
Supplement: Supplementary file 1 [file microorganisms-09-00438-s001.zip › Supplemetary/Figure_S1.pdf]

A. CCHFV S segment nucleotide percentage identities. Complete ORFs were aligned and pairwise percentage identities were calculated using CLUSTAL Omega.

	DQ144418_DRC_1956	DQ076413_UGA_1958	**Mbarara_UGA_2019**	MH178082_UGA_2015	MF511221_ZAF_1985	DQ211640_SEN_1972	DQ211639_SEN_1979	U88410_NGA_1966	DQ211647_ZAF_1987	DQ211646_ZAF_1985	MF547415_ESP_2014	DQ211641_MRT_1984	MF287636_ESP_2016	HQ378179_SDN_2009	GQ862371_SDN_2008	KX238958_NGA_2012	DQ076416_ZAF_1981	EF123122_CAF_1975	KX013452_NGA_1965	AF481802_RUS_2000	DQ211643_RUS_1967	DQ206447_RUS_2002	AY277672_RUS_2000	KR092375_TUR_2012	GU477489_BGR_1981	KJ027522_IRN_2013	DQ211649_TUR_2003	AF428144_KOS_2001	DQ133507_KOS_2001	MN866203_IND_2019	DQ211645_OMN_1997	MG659724_CHN_2017	AF527810_PAK_1976	U88414_PAK_1965	HM452305_AFG_2009	DQ446212_IRN_1952	AY223475_UZB_1967	AY049083_TJK_1990	JF922674_IND_2011	FJ562093_CHN_2004	GU477494_CHN_2004	AY297692_TJK_1990	AF481799_UZB_1985	U88413_CHN_1968	AJ010648_CHN_1966	DQ211638_GRC_1975	
DQ144418_DRC_1956		96	**93**	92	92	84	83	85	86	86	86	86	85	85	86	85	86	86	85	87	87	87	87	86	86	87	87	87	86	86	86	86	86	86	87	86	87	88	88	87	87	88	88	88	88	82	
DQ076413_UGA_1958	96		**92**	91	92	83	83	85	86	86	85	85	85	85	85	85	85	86	85	87	86	87	87	86	86	87	87	86	86	86	86	86	86	86	86	86	87	87	87	87	86	87	87	86	87	82	
**Mbarara_UGA_2019**	**93**	**92**		**94**	**94**	**84**	**84**	**85**	**86**	**86**	**86**	**85**	**85**	**85**	**85**	**85**	**86**	**85**	**85**	**86**	**86**	**86**	**86**	**86**	**86**	**86**	**86**	**86**	**86**	**86**	**86**	**86**	**86**	**87**	**87**	**86**	**87**	**88**	**87**	**87**	**86**	**87**	**87**	**87**	**87**	**82**	**Africa 2 (II)**
MH178082_UGA_2015	92	91	**94**		94	84	84	85	86	86	85	85	85	85	85	85	85	85	85	86	87	87	87	87	86	86	86	87	87	86	86	87	86	87	87	86	87	87	87	87	86	87	87	87	88	83	
MF511221_ZAF_1985	92	92	**94**	94		83	83	85	86	86	85	85	85	85	85	85	86	85	85	86	87	87	86	86	87	87	87	87	87	86	87	87	86	86	86	86	88	88	88	87	87	88	88	88	88	82	
DQ211640_SEN_1972	84	83	**84**	84	83		99	83	84	84	84	84	84	84	83	84	84	82	83	83	83	83	83	84	84	84	84	84	84	83	84	84	84	85	85	85	84	84	83	84	84	84	84	84	84	83	**Africa 1 (I)**
DQ211639_SEN_1979	83	83	**84**	84	83	99		83	83	84	84	84	84	84	83	84	83	83	83	83	83	83	83	84	84	84	84	84	84	83	84	84	84	84	85	84	84	84	84	84	84	84	84	84	84	83	
U88410_NGA_1966	85	85	**85**	85	85	83	83		93	93	92	92	92	92	92	93	93	86	86	86	86	86	86	86	86	86	86	87	87	88	88	87	88	88	89	88	87	88	88	89	88	88	88	88	89	83	
DQ211647_ZAF_1987	86	86	**86**	86	86	84	83	93		98	94	94	94	94	94	95	95	86	86	87	87	87	87	86	87	87	87	87	87	89	89	88	89	89	89	89	88	88	88	89	89	89	89	89	89	83	
DQ211646_ZAF_1985	86	86	**86**	86	86	84	84	93	98		94	94	94	94	94	95	95	86	86	87	86	87	86	86	86	87	87	87	87	89	89	89	89	89	89	89	88	88	88	89	88	88	89	89	89	83	
MF547415_ESP_2014	86	85	**86**	85	85	84	84	92	94	94		99	99	99	96	96	96	86	86	87	87	87	87	87	87	87	87	87	87	89	88	88	88	89	89	89	88	88	87	88	88	88	88	89	88	82	
DQ211641_MRT_1984	86	85	**85**	85	85	84	84	92	94	94	99		99	99	96	96	96	86	86	86	87	86	86	86	86	87	87	87	87	89	88	88	88	89	89	89	88	88	87	88	87	88	88	89	88	82	**Africa 3 (IIIa)**
MF287636_ESP_2016	85	85	**85**	85	85	84	84	92	94	94	99	99		99	96	96	96	86	86	86	86	86	86	86	86	87	87	86	86	88	88	88	88	89	89	89	87	88	87	88	87	88	88	88	88	82	
HQ378179_SDN_2009	85	85	**85**	85	85	84	84	92	94	94	99	99	99		96	96	96	86	86	86	86	86	86	86	86	86	86	86	86	88	88	88	88	88	89	88	87	87	87	88	87	87	88	88	88	82	
GQ862371_SDN_2008	86	85	**85**	85	85	83	83	92	94	94	96	96	96	96		97	97	86	86	87	87	87	87	87	87	88	87	88	88	89	89	88	89	89	89	89	88	88	87	89	88	88	89	89	89	83	
KX238958_NGA_2012	85	85	**85**	85	85	84	84	93	95	95	96	96	96	96	97		98	86	86	87	87	87	87	87	87	87	87	87	87	88	89	88	89	89	89	89	88	88	88	89	88	89	89	89	89	82	
DQ076416_ZAF_1981	86	85	**86**	85	86	84	83	93	95	95	96	96	96	96	97	98		86	86	87	87	87	87	87	87	87	87	87	87	89	89	88	89	89	90	90	88	88	88	89	88	89	89	89	89	83	
EF123122_CAF_1975	86	86	**85**	85	85	82	83	86	86	86	86	86	86	86	86	86	86		99	87	87	88	87	87	87	87	87	88	88	89	89	88	89	89	89	89	88	89	88	89	89	89	89	89	90	82	**Africa 4 (IIIb)**
KX013452_NGA_1965	85	85	**85**	85	85	83	83	86	86	86	86	86	86	86	86	86	86	99		87	87	87	87	87	87	87	87	87	87	88	89	88	89	89	89	89	88	89	88	89	89	89	89	89	89	82	
AF481802_RUS_2000	87	87	**86**	86	86	83	83	86	87	87	87	86	86	86	87	87	87	87	87		98	99	99	97	97	98	98	98	97	89	89	89	89	89	89	89	89	89	89	90	89	90	89	90	89	83	
DQ211643_RUS_1967	87	86	**86**	87	87	83	83	86	87	86	87	87	86	86	87	87	87	87	87	98		99	99	97	97	98	98	98	97	89	89	89	89	89	89	89	88	89	89	90	89	89	89	90	89	83	
DQ206447_RUS_2002	87	87	**86**	87	87	83	83	86	87	87	87	86	86	86	87	87	87	88	87	99	99		100	97	98	98	98	98	98	89	89	89	89	90	89	89	89	89	89	90	89	90	89	90	89	83	
AY277672_RUS_2000	87	87	**86**	87	86	83	83	86	87	86	87	86	86	86	87	87	87	87	87	99	99	100		97	97	98	98	98	98	89	89	89	89	89	89	89	89	89	89	89	89	90	89	90	89	83	
KR092375_TUR_2012	86	86	**86**	87	86	84	84	86	86	86	87	86	86	86	87	87	87	87	87	97	97	97	97		98	97	97	98	97	89	89	89	89	89	89	89	88	89	89	89	89	89	89	89	89	83	
GU477489_BGR_1981	86	86	**86**	86	87	84	84	86	87	86	87	86	86	86	87	87	87	87	87	97	97	98	97	98		98	98	98	98	88	89	88	88	89	89	88	88	89	89	89	88	90	89	89	89	83	**Europe 1 (V)**
KJ027522_IRN_2013	87	87	**86**	86	87	84	84	86	87	87	87	87	87	86	88	87	87	87	87	98	98	98	98	97	98		100	99	98	89	90	89	89	90	89	89	89	89	89	90	89	90	89	90	89	83	
DQ211649_TUR_2003	87	87	**86**	86	87	84	84	86	87	87	87	87	87	86	87	87	87	87	87	98	98	98	98	97	98	100		98	98	89	90	89	89	90	89	89	89	89	89	89	89	90	89	90	89	83	
AF428144_KOS_2001	87	86	**86**	87	87	84	84	87	87	87	87	87	86	86	88	87	87	88	87	98	98	98	98	98	98	99	98		99	89	90	89	89	90	89	89	89	90	89	90	89	90	89	90	89	83	
DQ133507_KOS_2001	86	86	**86**	87	87	84	84	87	87	87	87	87	86	86	88	87	87	88	87	97	97	98	98	97	98	98	98	99		89	89	89	89	89	89	89	89	90	90	90	89	90	89	90	89	84	
MN866203_IND_2019	86	86	**86**	86	86	83	83	88	89	89	89	89	88	88	89	88	89	89	88	89	89	89	89	89	88	89	89	89	89		95	94	95	95	95	95	90	90	90	91	91	90	90	91	91	83	
DQ211645_OMN_1997	86	86	**86**	86	87	84	84	88	89	89	88	88	88	88	89	89	89	89	89	89	89	89	89	89	89	90	90	90	89	95		96	95	96	96	95	91	91	91	91	91	91	90	91	91	83	
MG659724_CHN_2017	86	86	**86**	87	87	84	84	87	88	89	88	88	88	88	88	88	88	88	88	89	89	89	89	89	88	89	89	89	89	94	96		95	96	95	95	89	90	90	90	90	90	90	90	90	82	
AF527810_PAK_1976	86	86	**86**	86	86	84	84	88	89	89	88	88	88	88	89	89	89	89	89	89	89	89	89	89	88	89	89	89	89	95	95	95		99	99	98	90	91	91	92	92	91	91	92	91	83	**Asia 1 (IV)**
U88414_PAK_1965	86	86	**87**	87	86	85	84	88	89	89	89	89	89	88	89	89	89	89	89	89	89	90	89	89	89	90	90	90	89	95	96	96	99		99	99	90	91	91	92	92	91	91	92	91	83	
HM452305_AFG_2009	87	86	**87**	87	86	85	85	89	89	89	89	89	89	89	89	89	90	89	89	89	89	89	89	89	89	89	89	89	89	95	96	95	99	99		99	91	91	91	92	92	91	91	92	91	83	
DQ446212_IRN_1952	86	86	**86**	86	86	85	84	88	89	89	89	89	89	88	89	89	90	89	89	89	89	89	89	89	88	89	89	89	89	95	95	95	98	99	99		91	91	91	92	92	91	91	92	91	83	
AY223475_UZB_1967	87	87	**87**	87	88	84	84	87	88	88	88	88	87	87	88	88	88	88	88	89	88	89	89	88	88	89	89	89	89	90	91	89	90	90	91	91		98	98	94	94	94	93	94	94	83	
AY049083_TJK_1990	88	87	**88**	87	88	84	84	88	88	88	88	88	88	87	88	88	88	89	89	89	89	89	89	89	89	89	89	90	90	90	91	90	91	91	91	91	98		99	94	94	94	94	94	94	83	
JF922674_IND_2011	88	87	**87**	87	88	83	84	88	88	88	87	87	87	87	87	88	88	88	88	89	89	89	89	89	89	89	89	89	90	90	91	90	91	91	91	91	98	99		94	94	94	94	94	93	83	
FJ562093_CHN_2004	87	87	**87**	87	87	84	84	89	89	89	88	88	88	88	89	89	89	89	89	90	90	90	89	89	89	90	89	90	90	91	91	90	92	92	92	92	94	94	94		98	95	95	95	94	84	
GU477494_CHN_2004	87	86	**86**	86	87	84	84	88	89	88	88	87	87	87	88	88	88	89	89	89	89	89	89	89	88	89	89	89	89	91	91	90	92	92	92	92	94	94	94	98		95	95	95	94	83	**Asia 2 (IV)**
AY297692_TJK_1990	88	87	**87**	87	88	84	84	88	89	88	88	88	88	87	88	89	89	89	89	90	89	90	90	89	90	90	90	90	90	90	91	90	91	91	91	91	94	94	94	95	95		97	96	96	83	
AF481799_UZB_1985	88	87	**87**	87	88	84	84	88	89	89	88	88	88	88	89	89	89	89	89	89	89	89	89	89	89	89	89	89	89	90	90	90	91	91	91	91	93	94	94	95	95	97		97	96	83	
U88413_CHN_1968	88	86	**87**	87	88	84	84	88	89	89	89	89	88	88	89	89	89	89	89	90	90	90	90	89	89	90	90	90	90	91	91	90	92	92	92	92	94	94	94	95	95	96	97		98	84	
AJ010648_CHN_1966	88	87	**87**	88	88	84	84	89	89	89	88	88	88	88	89	89	89	90	89	89	89	89	89	89	89	89	89	89	89	91	91	90	91	91	91	91	94	94	93	94	94	96	96	98		84	
DQ211638_GRC_1975	82	82	**82**	83	82	83	83	83	83	83	82	82	82	82	83	82	83	82	82	83	83	83	83	83	83	83	83	83	84	83	83	82	83	83	83	83	83	83	83	84	83	83	83	84	84		**Europe 2 (VI)**
	**Africa 2 (II)**					**Africa 1 (I)**		**Africa 3 (IIIa)**										**Africa 4 (IIIb)**		**Europe 1 (V)**										**Asia 1 (IV)**							**Asia 2 (IV)**									**Europe 2 (VI)**	

B. CCHFV M segment nucleotide percentage identities. Complete ORFs were aligned and pairwise percentage identities was calculated using CLUSTAL Omega.

	**Mbarara_UGA_2019**	DQ094832_UGA_1958	DQ211637_DRC_1956	MH688498_CHN_2016	FJ562094_CHN_2004	AB069673_CHN_1979	MH396665_IND_2016	MN866218_IND_2019	DQ211626_SEN_1969	DQ211627_SEN_1972	GU477490_BGR_1981	DQ206448_RUS_2002	DQ211636_TUR_2003	GQ337054_TUR_2006	EU037902_KOS_2001	DQ211630_RUS_1967	DQ211634_ZAF_1987	MF547416_ESP_2014	HQ378187_SDN_2009	MF287637_ESP_2016	KX013451_NGA_1965	KY484035_NGA_1996	AF467768_NGA_1966	DQ157174_ZAF_1981	HQ378185_SDN_2008	JN572086_IND_2011	MN930418_IND_2019	HM452306_AFG_2009	DQ446216_IRN_2002	DQ446215_IRN_2002	MF289418_UAE_1998	MG659723_CHN_2017	AY223476_UZB_1967	AY179962_TJK_1990	AJ538197_IRQ_1976	AB069671_CHN_1975	AB069672_CHN_1978	KX013445_TJK_1969	KC344856_AFG_2012	DQ211635_ZAF_1985	DQ211633_ZAF_1985	DQ211632_OMN_1997	KJ682805_ZAF_2008	KJ682808_NAM_1988	AF467769_PAK_1976	AF350449_CHN_1984	DQ211629_CHN_1968	DQ211625_GRC_1975	DQ211628_MRT_1984	
**Mbarara_UGA_2019**		**78**	**78**	**80**	**80**	**80**	**80**	**80**	**79**	**78**	**70**	**71**	**71**	**70**	**70**	**70**	**70**	**70**	**70**	**70**	**70**	**70**	**70**	**70**	**70**	**70**	**70**	**70**	**71**	**70**	**70**	**71**	**71**	**70**	**71**	**70**	**70**	**70**	**70**	**70**	**70**	**70**	**70**	**70**	**70**	**70**	**70**	**71**	**70**	**Africa 2 (II)**
DQ094832_UGA_1958	**78**		93	80	80	80	81	81	80	80	70	71	70	70	71	70	70	70	70	70	70	70	70	70	69	70	71	70	70	70	70	70	70	70	70	70	70	69	70	70	70	70	70	70	69	70	70	71	69	
DQ211637_DRC_1956	**78**	93		81	81	81	81	81	80	80	70	70	70	70	71	70	70	70	71	70	70	71	71	70	70	71	70	70	70	70	70	71	70	70	70	70	70	70	70	70	71	70	70	70	70	71	70	71	70	
MH688498_CHN_2016	**80**	80	81		99	98	83	83	82	82	71	72	72	72	72	71	71	71	71	71	71	71	71	71	71	72	72	71	71	71	71	71	71	71	72	71	71	71	71	71	72	72	71	71	71	71	71	72	71	
FJ562094_CHN_2004	**80**	80	81	99		99	83	82	82	82	71	72	72	72	72	71	71	71	71	71	71	71	71	71	71	72	72	71	71	71	71	71	71	71	72	71	71	71	71	71	72	72	71	71	71	71	71	72	71	
AB069673_CHN_1979	**80**	80	81	98	99		83	82	82	82	71	72	72	72	72	71	71	71	71	71	71	71	71	71	71	72	72	71	71	71	71	71	71	71	72	71	71	71	71	71	72	72	71	71	71	71	71	72	71	
MH396665_IND_2016	**80**	81	81	83	83	83		99	83	83	71	71	71	71	71	71	71	71	71	71	71	71	71	71	70	71	71	70	71	70	70	71	70	70	71	70	70	70	71	71	71	71	71	71	71	70	70	72	71	
MN866218_IND_2019	**80**	81	81	83	82	82	99		83	83	71	71	71	71	71	70	70	70	70	70	71	71	71	70	70	71	70	70	70	70	70	71	70	70	71	70	70	70	71	71	71	71	71	70	71	70	70	72	71	
DQ211626_SEN_1969	**79**	80	80	82	82	82	83	83		98	71	72	71	71	71	71	71	71	71	71	71	71	71	71	71	71	71	71	71	71	71	71	71	71	71	71	70	71	71	70	70	70	71	71	70	70	70	71	70	**Africa 1 (I)**
DQ211627_SEN_1972	**78**	80	80	82	82	82	83	83	98		71	71	71	71	71	70	71	71	71	71	71	71	71	71	70	71	71	71	71	71	71	71	71	70	71	70	70	70	70	70	70	70	70	70	70	70	70	71	70	
GU477490_BGR_1981	**70**	70	70	71	71	71	71	71	71	71		94	94	94	93	93	80	80	80	80	80	81	81	80	80	81	80	80	80	80	80	80	80	80	80	80	80	80	80	81	81	80	80	80	80	80	80	71	72	**Europe 1 (V)**
DQ206448_RUS_2002	**71**	71	70	72	72	72	71	71	72	71	94		95	95	95	94	81	80	81	81	81	81	81	81	80	81	81	81	81	81	81	81	81	81	81	81	81	81	81	82	82	81	81	80	81	81	81	71	72	
DQ211636_TUR_2003	**71**	70	70	72	72	72	71	71	71	71	94	95		99	94	94	81	80	81	81	81	81	81	81	80	81	81	81	81	81	81	81	81	80	81	81	81	81	81	81	81	81	81	80	81	81	81	71	72	
GQ337054_TUR_2006	**70**	70	70	72	72	72	71	71	71	71	94	95	99		94	94	81	80	81	81	81	81	81	81	80	81	81	81	81	81	81	80	81	80	80	81	80	81	81	81	81	81	80	80	81	81	81	71	72	
EU037902_KOS_2001	**70**	71	71	72	72	72	71	71	71	71	93	95	94	94		95	81	81	81	81	81	81	81	81	80	81	81	81	80	81	81	81	81	80	81	81	80	81	81	81	81	81	80	80	81	81	81	71	72	
DQ211630_RUS_1967	**70**	70	70	71	71	71	71	70	71	70	93	94	94	94	95		80	80	81	81	81	81	81	81	80	81	80	81	80	81	81	80	80	80	81	81	81	81	81	81	81	81	80	80	80	80	81	71	72	
DQ211634_ZAF_1987	**70**	70	70	71	71	71	71	70	71	71	80	81	81	81	81	80		91	91	91	91	91	91	92	91	88	87	88	88	88	88	88	88	87	87	89	88	80	81	81	81	80	81	81	80	80	81	71	72	**Africa 3 (III)**
MF547416_ESP_2014	**70**	70	70	71	71	71	71	70	71	71	80	80	80	80	81	80	91		97	96	97	97	97	95	94	88	87	87	87	87	88	88	88	87	87	89	88	81	81	81	81	81	81	81	81	81	81	71	72	
HQ378187_SDN_2009	**70**	70	71	71	71	71	71	70	71	71	80	81	81	81	81	81	91	97		99	97	98	98	95	94	88	87	87	88	87	88	88	88	87	88	89	89	81	80	81	81	82	80	80	81	81	81	71	72	
MF287637_ESP_2016	**70**	70	70	71	71	71	71	70	71	71	80	81	81	81	81	81	91	96	99		97	98	98	96	94	88	87	88	88	88	88	89	88	88	88	89	89	81	80	81	81	81	80	80	81	81	81	71	72	
KX013451_NGA_1965	**70**	70	70	71	71	71	71	71	71	71	80	81	81	81	81	81	91	97	97	97		98	98	96	94	88	87	87	87	87	88	88	88	87	88	89	89	80	81	81	81	81	80	80	81	81	81	71	72	
KY484035_NGA_1996	**70**	70	71	71	71	71	71	71	71	71	81	81	81	81	81	81	91	97	98	98	98		100	96	95	88	87	88	88	88	88	89	89	88	88	89	89	81	81	81	81	81	81	80	81	81	81	71	72	
AF467768_NGA_1966	**70**	70	71	71	71	71	71	71	71	71	81	81	81	81	81	81	91	97	98	98	98	100		96	95	88	87	88	88	88	88	89	89	88	88	89	89	81	81	81	81	81	80	80	81	81	81	71	72	
DQ157174_ZAF_1981	**70**	70	70	71	71	71	71	70	71	71	80	81	81	81	81	81	92	95	95	96	96	96	96		96	88	88	88	88	88	88	89	89	88	88	90	89	81	80	81	81	81	80	80	81	80	81	71	72	
HQ378185_SDN_2008	**70**	69	70	71	71	71	70	70	71	70	80	80	80	80	80	80	91	94	94	94	94	95	95	96		88	87	87	87	87	87	88	88	87	87	89	89	81	80	81	80	81	80	80	80	80	80	71	72	
JN572086_IND_2011	**70**	70	71	72	72	72	71	71	71	71	81	81	81	81	81	81	88	88	88	88	88	88	88	88	88		92	93	93	93	93	89	89	88	88	89	89	81	81	81	82	81	81	81	82	81	82	72	72	**Asia 1 (IV)**
MN930418_IND_2019	**70**	71	70	72	72	72	71	70	71	71	80	81	81	81	81	80	87	87	87	87	87	87	87	88	87	92		94	94	94	94	88	88	88	88	89	89	81	80	80	80	81	80	80	81	80	80	72	72	
HM452306_AFG_2009	**70**	70	70	71	71	71	70	70	71	71	80	81	81	81	81	81	88	87	87	88	87	88	88	88	87	93	94		99	99	99	89	89	88	88	89	89	81	80	81	81	81	81	80	81	81	81	72	72	
DQ446216_IRN_2002	**71**	70	70	71	71	71	71	70	71	71	80	81	81	81	80	80	88	87	88	88	87	88	88	88	87	93	94	99		100	99	89	89	88	88	89	89	81	80	81	81	81	81	80	81	81	81	72	72	
DQ446215_IRN_2002	**70**	70	70	71	71	71	70	70	71	71	80	81	81	81	81	81	88	87	87	88	87	88	88	88	87	93	94	99	100		99	89	89	88	88	89	89	81	80	81	81	81	81	80	81	81	81	72	72	
MF289418_UAE_1998	**70**	70	70	71	71	71	70	70	71	71	80	81	81	81	81	81	88	88	88	88	88	88	88	88	87	93	94	99	99	99		89	89	88	88	89	89	81	80	81	81	81	81	80	81	81	81	72	72	
MG659723_CHN_2017	**71**	70	71	71	71	71	71	71	71	71	80	81	81	80	81	80	88	88	88	89	88	89	89	89	88	89	88	89	89	89	89		92	92	92	93	93	81	80	81	81	81	81	80	81	81	81	72	72	
AY223476_UZB_1967	**71**	70	70	71	71	71	70	70	71	71	80	81	81	81	81	80	88	88	88	88	88	89	89	89	88	89	88	89	89	89	89	92		96	93	94	94	81	81	81	82	81	81	81	82	82	82	72	72	
AY179962_TJK_1990	**70**	70	70	71	71	71	70	70	71	70	80	81	80	80	80	80	87	87	87	88	87	88	88	88	87	88	88	88	88	88	88	92	96		92	94	94	81	80	81	81	81	80	80	81	81	81	72	72	
AJ538197_IRQ_1976	**71**	70	70	72	72	72	71	71	71	71	80	81	81	80	81	81	87	87	88	88	88	88	88	88	87	88	88	88	88	88	88	92	93	92		95	95	81	81	81	81	81	81	80	81	81	82	71	72	
AB069671_CHN_1975	**70**	70	70	71	71	71	70	70	71	70	80	81	81	81	81	81	89	89	89	89	89	89	89	90	89	89	89	89	89	89	89	93	94	94	95		99	81	80	82	82	81	81	81	81	81	82	71	72	
AB069672_CHN_1978	**70**	70	70	71	71	71	70	70	70	70	80	81	81	80	80	81	88	88	89	89	89	89	89	89	89	89	89	89	89	89	89	93	94	94	95	99		81	80	81	81	81	81	81	81	81	81	71	72	
KX013445_TJK_1969	**70**	69	70	71	71	71	70	70	71	70	80	81	81	81	81	81	80	81	81	81	80	81	81	81	81	81	81	81	81	81	81	81	81	81	81	81	81		84	83	83	84	83	83	84	84	84	71	72	**Asia 2 (IV)**
KC344856_AFG_2012	**70**	70	70	71	71	71	71	71	71	70	80	81	81	81	81	81	81	81	80	80	81	81	81	80	80	81	80	80	80	80	80	80	81	80	81	80	80	84		84	84	84	83	83	84	84	84	71	72	
DQ211635_ZAF_1985	**70**	70	70	71	71	71	71	71	70	70	81	82	81	81	81	81	81	81	81	81	81	81	81	81	81	81	80	81	81	81	81	81	81	81	81	82	81	83	84		98	86	86	86	87	87	87	71	72	
DQ211633_ZAF_1985	**70**	70	71	72	72	72	71	71	70	70	81	82	81	81	81	81	81	81	81	81	81	81	81	81	80	82	80	81	81	81	81	81	82	81	81	82	81	83	84	98		87	86	86	87	87	87	71	72	
DQ211632_OMN_1997	**70**	70	70	72	72	72	71	71	70	70	80	81	81	81	81	81	80	81	82	81	81	81	81	81	81	81	81	81	81	81	81	81	81	81	81	81	81	84	84	86	87		87	87	88	88	88	71	73	
KJ682805_ZAF_2008	**70**	70	70	71	71	71	71	71	71	70	80	81	81	80	80	80	81	81	80	80	80	81	80	80	80	81	80	81	81	81	81	81	81	80	81	81	81	83	83	86	86	87		98	90	90	90	71	72	
KJ682808_NAM_1988	**70**	70	70	71	71	71	71	70	71	70	80	80	80	80	80	80	81	81	80	80	80	80	80	80	80	81	80	80	80	80	80	80	81	80	80	81	81	83	83	86	86	87	98		90	90	90	71	72	
AF467769_PAK_1976	**70**	69	70	71	71	71	71	71	70	70	80	81	81	81	81	80	80	81	81	81	81	81	81	81	80	82	81	81	81	81	81	81	82	81	81	81	81	84	84	87	87	88	90	90		93	94	71	72	
AF350449_CHN_1984	**70**	70	71	71	71	71	70	70	70	70	80	81	81	81	81	80	80	81	81	81	81	81	81	80	80	81	80	81	81	81	81	81	82	81	81	81	81	84	84	87	87	88	90	90	93		97	71	72	
DQ211629_CHN_1968	**70**	70	70	71	71	71	70	70	70	70	80	81	81	81	81	81	81	81	81	81	81	81	81	81	80	82	80	81	81	81	81	81	82	81	82	82	81	84	84	87	87	88	90	90	94	97		71	72	
DQ211625_GRC_1975	**71**	71	71	72	72	72	72	72	71	71	71	71	71	71	71	71	71	71	71	71	71	71	71	71	71	72	72	72	72	72	72	72	72	72	71	71	71	71	71	71	71	71	71	71	71	71	71		71	**Europe 2 (VI)**
DQ211628_MRT_1984	**70**	69	70	71	71	71	71	71	70	70	72	72	72	72	72	72	72	72	72	72	72	72	72	72	72	72	72	72	72	72	72	72	72	72	72	72	72	72	72	72	72	73	72	72	72	72	72	71		**Mauritania (VII)**
	**Africa 2 (II)**								**Africa 1 (I)**		**Europe 1 (V)**						**Africa 3 (III)**									**Asia 1 (IV)**												**Asia 2 (IV)**										**Europe 2 (VI)**	**Mauritania (VII)**	

C. CCHFV L segment nucleotide percentage identities. Complete ORFs were aligned and pairwise percentage identities was calculated using CLUSTAL Omega.

	MF511204_ZAF_1985	**Mbarara_UGA_2019**	DQ211624_DRC_1956	DQ076412_UGA_1958	KX013483_UGA_1958	MN930408_IND_2019	AY720893_TJK_1990	KX013447_UZB_1967	DQ211619_OMN_1997	MG659722_CHN_2017	JN627865_IND_2011	HM452307_AFG_2009	AY422208_PAK_1976	AY947890_IRQ_1979	FJ562095_CHN_2004	GU477492_CHN_2004	DQ211623_TUR_2003	GQ337055_TUR_2006	EU044832_KOS_2001	DQ211617_RUS_1967	AY675240_RUS_2002	AY995166_RUS_2003	DQ211618_RUS_1967	DQ211613_SEN_1969	DQ211614_SEN_1972	KX013450_NGA_1965	DQ076417_ZAF_1981	DQ211622_ZAF_1985	DQ211620_ZAF_1985	MF547417_ESP_2014	DQ211615_MRT_1984	MF287638_ESP_2016	KX238956_NGA_2012	HQ378183_SDN_2009	AY422209_NGA_1966	DQ211621_ZAF_1987	MF511201_NAM_1987	HQ378180_SDN_2008	DQ211612_GRC_1975	
MF511204_ZAF_1985		92	92	92	92	84	84	84	84	84	84	85	84	84	85	85	84	84	84	84	84	84	84	84	84	84	84	84	84	84	84	84	84	84	84	84	84	84	78	
**Mbarara_UGA_2019**	92		94	94	94	84	84	84	84	84	84	84	84	84	85	85	84	84	84	84	84	84	84	84	83	83	83	84	84	84	83	84	83	84	84	84	83	84	78	
DQ211624_DRC_1956	92	94		96	94	84	84	84	84	84	84	84	84	84	85	85	84	84	84	84	84	84	84	84	83	83	83	84	84	83	83	84	83	84	83	84	83	84	78	Africa 2 (II)
DQ076412_UGA_1958	92	94	96		98	84	84	84	84	84	84	85	84	84	85	85	84	84	84	84	84	84	84	84	83	84	83	84	84	84	83	83	83	84	84	84	83	84	78	
KX013483_UGA_1958	92	94	94	98		84	84	84	84	84	84	84	84	84	84	85	84	84	84	84	84	84	84	84	83	83	83	83	84	83	83	83	83	83	83	83	83	83	78	
MN930408_IND_2019	84	84	84	84	84		97	97	88	88	89	89	89	89	88	88	88	87	88	88	88	88	88	87	87	87	87	87	87	87	87	87	87	87	87	87	87	86	78	
AY720893_TJK_1990	84	84	84	84	84	97		98	88	88	89	89	89	89	88	88	88	88	88	88	88	88	88	87	87	86	87	87	87	87	87	87	87	87	87	87	87	86	78	Asia 1 (IV)
KX013447_UZB_1967	84	84	84	84	84	97	98		88	88	89	89	89	89	88	88	88	88	88	88	88	88	88	87	87	86	87	87	87	87	87	87	87	87	87	87	87	86	78	
DQ211619_OMN_1997	84	84	84	84	84	88	88	88		92	92	92	92	92	87	87	87	87	87	87	87	87	87	87	86	86	86	87	86	86	86	86	86	86	87	87	86	86	79	
MG659722_CHN_2017	84	84	84	84	84	88	88	88	92		95	95	92	92	88	88	87	87	87	87	87	87	87	86	86	86	86	86	86	86	86	86	86	86	86	87	87	86	79	
JN627865_IND_2011	84	84	84	84	84	89	89	89	92	95		98	92	92	88	88	87	87	87	88	88	88	88	87	87	86	87	87	87	87	87	87	87	87	87	87	87	87	79	
HM452307_AFG_2009	85	84	84	85	84	89	89	89	92	95	98		92	92	88	88	87	87	88	88	88	88	88	87	87	86	87	87	87	87	87	87	87	87	87	87	87	87	79	Asia 1 (IV)
AY422208_PAK_1976	84	84	84	84	84	89	89	89	92	92	92	92		92	88	88	87	87	87	88	87	87	87	87	87	86	86	87	87	87	87	87	87	87	87	87	87	87	79	
AY947890_IRQ_1979	84	84	84	84	84	89	89	89	92	92	92	92	92		88	88	87	87	88	88	88	88	88	87	87	86	87	87	87	87	87	87	87	87	87	88	87	87	78	
FJ562095_CHN_2004	85	85	85	85	84	88	88	88	87	88	88	88	88	88		99	88	88	89	89	88	88	88	88	87	87	88	88	88	88	88	88	87	88	88	88	88	88	78	Asia 3 (IV)
GU477492_CHN_2004	85	85	85	85	85	88	88	88	87	88	88	88	88	88	99		89	89	89	89	88	89	89	88	88	87	88	88	88	88	88	88	88	88	88	88	88	88	78	
DQ211623_TUR_2003	84	84	84	84	84	88	88	88	87	87	87	87	87	87	88	89		99	96	96	96	96	96	90	90	89	89	90	90	90	90	90	90	90	90	90	90	89	78	
GQ337055_TUR_2006	84	84	84	84	84	87	88	88	87	87	87	87	87	87	88	89	99		96	96	96	96	96	89	89	89	89	90	89	89	90	90	89	90	90	90	90	89	78	
EU044832_KOS_2001	84	84	84	84	84	88	88	88	87	87	87	88	87	88	89	89	96	96		97	96	96	97	90	90	89	90	90	90	90	90	90	90	90	90	90	90	89	78	
DQ211617_RUS_1967	84	84	84	84	84	88	88	88	87	87	88	88	88	88	89	89	96	96	97		98	99	99	90	89	89	89	90	90	90	90	90	90	90	90	90	90	89	78	Europe 1 (V)
AY675240_RUS_2002	84	84	84	84	84	88	88	88	87	87	88	88	87	88	88	88	96	96	96	98		99	99	90	89	89	89	90	90	89	90	90	90	90	90	90	90	89	78	
AY995166_RUS_2003	84	84	84	84	84	88	88	88	87	87	88	88	87	88	88	89	96	96	96	99	99		100	90	89	89	90	90	90	90	90	90	90	90	90	90	90	89	78	
DQ211618_RUS_1967	84	84	84	84	84	88	88	88	87	87	88	88	87	88	88	89	96	96	97	99	99	100		90	89	89	90	90	90	90	90	90	90	90	90	90	90	89	78	
DQ211613_SEN_1969	84	84	84	84	84	87	87	87	87	86	87	87	87	87	88	88	90	89	90	90	90	90	90		98	92	93	95	95	98	98	98	98	98	98	93	94	93	78	Africa 1 (I)
DQ211614_SEN_1972	84	83	83	83	83	87	87	87	86	86	87	87	87	87	87	88	90	89	90	89	89	89	89	98		92	93	95	95	98	98	98	98	98	98	93	93	93	78	
KX013450_NGA_1965	84	83	83	84	83	87	86	86	86	86	86	86	86	86	87	87	89	89	89	89	89	89	89	92	92		92	92	92	92	92	92	92	92	92	93	93	92	78	
DQ076417_ZAF_1981	84	83	83	83	83	87	87	87	86	86	87	87	86	87	88	88	89	89	90	89	89	90	90	93	93	92		93	93	93	93	93	93	93	93	93	93	92	78	
DQ211622_ZAF_1985	84	84	84	84	83	87	87	87	87	86	87	87	87	87	88	88	90	90	90	90	90	90	90	95	95	92	93		99	95	95	95	95	95	95	93	93	93	78	
DQ211620_ZAF_1985	84	84	84	84	84	87	87	87	87	86	87	87	87	87	88	88	90	89	90	90	90	90	90	95	95	92	93	99		95	95	95	95	95	95	93	93	93	78	
MF547417_ESP_2014	84	84	83	84	83	87	87	87	86	86	87	87	87	87	88	88	90	89	90	90	89	90	90	98	98	92	93	95	95		99	98	98	98	98	93	93	93	78	
DQ211615_MRT_1984	84	83	83	83	83	87	87	87	86	86	87	87	87	87	88	88	90	90	90	90	90	90	90	98	98	92	93	95	95	99		99	99	99	98	93	93	93	78	
MF287638_ESP_2016	84	84	84	83	83	87	87	87	86	86	87	87	87	87	88	88	90	90	90	90	90	90	90	98	98	92	93	95	95	98	99		99	99	98	93	94	93	78	Africa 3 (III)
KX238956_NGA_2012	84	83	83	83	83	87	87	87	86	86	87	87	87	87	87	88	90	89	90	90	90	90	90	98	98	92	93	95	95	98	99	99		99	98	93	93	93	78	
HQ378183_SDN_2009	84	84	84	84	83	87	87	87	86	86	87	87	87	87	88	88	90	90	90	90	90	90	90	98	98	92	93	95	95	98	99	99	99		98	93	94	93	79	
AY422209_NGA_1966	84	84	83	84	83	87	87	87	87	86	87	87	87	87	88	88	90	90	90	90	90	90	90	98	98	92	93	95	95	98	98	98	98	98		93	93	93	78	
DQ211621_ZAF_1987	84	84	84	84	83	87	87	87	87	87	87	87	87	88	88	88	90	90	90	90	90	90	90	93	93	93	93	93	93	93	93	93	93	93	93		94	93	79	
MF511201_NAM_1987	84	83	83	83	83	87	87	87	86	87	87	87	87	87	88	88	90	90	90	90	90	90	90	94	93	93	93	93	93	93	93	94	93	94	93	94		94	78	
HQ378180_SDN_2008	84	84	84	84	83	86	86	86	86	86	87	87	87	87	88	88	89	89	89	89	89	89	89	93	93	92	92	93	93	93	93	93	93	93	93	93	94		78	
DQ211612_GRC_1975	78	78	78	78	78	78	78	78	79	79	79	79	79	78	78	78	78	78	78	78	78	78	78	78	78	78	78	78	78	78	78	78	78	79	78	79	78	78		Europe 2 (VI)
			Africa 2 (II)				Asia 1 (IV)				Asia 2 (IV)				Asia 3 (IV)					Europe 1 (V)				Africa 1 (I)								Africa 3 (III)							Europe 2 (VI)	
